# The Influence of Red Cabbage Extract Nanoencapsulated with Brassica Plasma Membrane Vesicles on the Gut Microbiome of Obese Volunteers

**DOI:** 10.3390/foods10051038

**Published:** 2021-05-10

**Authors:** Paula Garcia-Ibañez, Carles Roses, Agatha Agudelo, Fermin I. Milagro, Ana M. Barceló, Blanca Viadel, Juan Antonio Nieto, Diego A. Moreno, Micaela Carvajal

**Affiliations:** 1 Aquaporins Group, Centro de Edafología y Biología Aplicada del Segura, CEBAS-CSIC, Campus Universitario de Espinardo-25, E-30100 Murcia, Spain; pgibanez@cebas.csic.es (P.G.-I.); mcarvaja@cebas.csic.es (M.C.); 2 Phytochemistry and Healthy Foods Lab, Department of Food Science Technology, Centro de Edafología y Biología Aplicada del Segura, CEBAS-CSIC, Campus de Espinardo-25, E-30100 Murcia, Spain; 3 Servei de Genòmica I Bioinformàtica, Universitat Autònoma de Barcelona, 08193 Bellaterra, Spain; crosespol@gmail.com (C.R.); anna.barcelo@uab.cat (A.M.B.); 4 Sakata Seed Ibérica S.L., Pl. Poeta Vicente Gaos, 6 Bajo, 46021 Valencia, Spain; agatha.agudelo@sakata.eu; 5 Biotechnology Department, Universidad Politécnica de Valencia, UPV, Camino de Vera s/n, 46022 Valencia, Spain; 6 Center for Nutrition Research, Department of Nutrition, Food Sciences and Physiology, University of Navarra, 31008 Pamplona, Spain; fmilagro@unav.es; 7 Navarra Institute for Health Research (IdISNA), 31008 Pamplona, Spain; 8 Centro de Investigación Biomédica en Red de la Fisiopatología de la Obesidad y Nutrición (CIBERobn), Instituto de la Salud Carlos III, 289029 Madrid, Spain; 9 AINIA, Technology Centre, C/Benjamin Franklin 5-11, Parque Tecnológico de Valencia, 46980 Paterna, Valencia, Spain; bviadel@ainia.es (B.V.); jnieto@ainia.es (J.A.N.)

**Keywords:** red cabbage, isothiocyanates, stability, encapsulation, gut microbiome, obesity

## Abstract

The aim of the study was to evaluate the influence of the red cabbage extracts on the bioaccessibility of their isothiocyanates, and their effect on the intestinal microbiota using a dynamic model of human digestion treated with the gut microbiome of obese adults. The elicitation of red cabbage plants with methyl jasmonate (MeJA) duplicated the content of glucosinolates (GSLs) in the plant organs used for elaborating the encapsulated formula. The use of plasma membrane vesicles, according to a proper methodology and technology, showed a high retention of sulforaphane (SFN) and indol-3-carbinol (I3C) over the course of the 14-day digestion study. The microbiome was scarcely affected by the treatments in terms of microbiota composition or the *Bacteroidetes/Firmicutes* ratio, but a 3 to 4-fold increase was observed in the production of butyric acid with the encapsulated extract treatment. Based on our pilot red cabbage extract study, the consumption of this extract, mainly encapsulated, may play a potential role in the management of obesity in adults.

## 1. Introduction

Brassica vegetables stand out as dietary coadjutants, functional ingredient sources, or natural bioactive-rich foods, and their consumption may result in a healthier life or prevent some illnesses [1,2], including the modulation of inflammation in overweight and obese adults (a BMI ≥ 25 for overweight subjects, and a BMI of 29.9–34.9 for obese subjects) [3]. Particularly, red cabbage (*Brassica oleracea* L. var. *capitata* f. *rubra*) has been studied as a source of health-promoting phytochemicals, including glucosinolates (GSLs) and different classes of phenolic compounds, such as acylated anthocyanins, flavonol glycosides, and hydroxycinnamic acid derivatives [4,5]. GSLs are stable in their natural matrix, but after tissue disruption, they are hydrolyzed by myrosinase (EC 3.2.1.147), resulting in the production of bioavailable and bioactive compounds, namely, isothiocyanates (ITCs) and indoles [6]. These have been classically studied as the compounds responsible for the effects on Phase II detoxification enzymes, which have anti-tumorigenic effects [7]. More recently, the bioactive compounds of the *Brassica* species have been associated with the modulation of the gastrointestinal microbiome [8,9].

In order to increase the phytochemical content in plant-derived foods and products, the use of elicitation has emerged as a useful tool [10]. The content of glucosinolates in cruciferous plants and sprouts can be manipulated through treatments with elicitors, such as plant hormones (methyl jasmonate (MeJA), jasmonic acid (JA), salicylic acid (SA), ethylene (ET), or abscisic acid (ABA), among others), or mineral or amino acid solutions [11]. These various compounds act as stressors in the plants, and activate an array of mechanisms similar to the defense responses to pathogen infections or environmental stimuli, ultimately affecting the plant’s metabolic pathways by enhancing the synthesis of phytochemicals [12]. Jasmonic acid and its methyl ester (MeJA), and methionine, were applied to broccoli and radish sprouts [13] to increase the individual and total contents of GSLs. More recently for Bimi^®^ (a hybrid between broccoli and green Chinese kale) plants in field experiments, the content of GSLs was also increased by the application of 100 μmol L^−1^ of MeJA [14]. SA is a widely used elicitor ^15^, and it has been reported that it increases the contents of GSLs in broccoli, China rose radish, and red radish sprouts [15,16]. The evidence on the use of elicitors for the enrichment of plant-derived products are widely available in literature [13,14,17].

Presently, a global trend has been observed on the demand for highly nutritious and easy-to-consume-foods, including functional foods or beverages [18]. Different sources of ingredients from *Brassica* species have been used to date [14,19], which are usually prepared as extracts rich in ITCs [20,21]. The problems with using ITCs as ingredients include their low water solubility and very limited stability [22,23], when used in nutritional interventions and compared to the intake of GSLs from a natural matrix (e.g., broccoli sprouts) [24]. With the aim of increasing the stability and bioavailability of ITCs, the use of encapsulation could provide a solution [25]. Recently, the use of plant plasma membrane vesicles as delivery systems for bioactive compounds has been studied [26,27]. Diverse types of plant membrane vesicles could be good candidates for this purpose, such as extracellular vesicles, which are spheroids of cytosolic material surrounded by a lipid bilayer, or extracted plasma membrane from fresh plant tissue [28]. As an example of the latter, we used cauliflower plasma membrane vesicles, which are proteoliposomes with a high proportion of unsaturated fatty acids. Furthermore, the proteomic analysis performed demonstrated the presence of aquaporins, such as PIP1 or PIP2, which grant a high osmotic permeability to this vesicles [29]. However, the information of the performance of these encapsulated formulas in the GI tract is rather limited [30], and one of the objectives of this work was to evaluate this possibility.

The current trends and demands for a healthier diet include consuming more vegetables and fruits on a daily basis [31]. Worldwide national health systems recommend such improvements in diets in order to help counteract the high prevalence of metabolic-related diseases, such as obesity, diabetes, or hyperglycemia [32,33,34], along with economic and social considerations [35,36]. Nevertheless, these recommendations have not achieved the expected impact, and this situation has led to one of the major causes of death globally [37]. Only recently, the role of the gut microbiome in this context has taken the main stage, and reports on the association between alterations in the gut microbiome (dysbiosis), and modifications in the gut barrier permeability [38], have been shown to be present in obese subjects with a dysbiotic gut microbiome [39]. Furthermore, associations between the gut microbiome and energy imbalances have also been found in obesity [40].

Therefore, the aims of this work were to determine the influence of the elicitation method (MeJA, SA, or its combination) on the content of glucosinolates in red cabbage, in order to use the plant material as a source of bioactive ITCs; to improve the stability of these ITCs by means of nanoencapsulation of red cabbage-derived aqueous extracts; and, to evaluate their bioaccessibility and their effect on the intestinal microbiota, by using an in vitro dynamic gastrointestinal system, conditioned with gut microbiome from obese adults.

The characterisation of the composition of the red cabbage, the derived extracts, and the samples obtained from the digestion system were carried out to establish the basis for future food product developments for the management of obesity.

## 2. Materials and Methods

### 2.1. Plant Material and Treatments

One hundred ninety-two red cabbage (*Brassica oleracea* L. var. capitata f. rubra) seeds from Sakata Seed Iberica (Valencia, Spain) were pre-treated with deionized water and continuous aeration for 24 h. Then, seeds were planted in vermiculite for 2 days in darkness and at 28 °C and 60% relative humidity. Seedlings were transplanted to experimental soil in a farm (37°47′52.7″ N, 0°52′00.7″ W, 15 m asl, Murcia, Spain). The plants were grown from September 2018 to February 2019 under a semiarid Mediterranean climate. Plants were drip irrigated with ¼ Hoagland solution. Twenty-four plants were assigned to each treatment, with two replicates. Elicitation treatments (150 mL of solution sprayed per plant, using an elicitation backpack) were as follows: (i) control, 0.2% ethanol; (ii) 100 μM MeJA in 0.2% ethanol; (iii) 200 μM SA in 0.2% ethanol; and iv) combined administration of SA + MeJA. The selection of dosages was based on previous experiments [14,16]. Treatments were also supplemented with a patented concentration of surfactant (Patent # PCT/ES2019/070457). The treatments were applied with the appearance of the flower bud and for 5 days the plants were kept growing, and 5 days after, a second round of 5 additional days of elicitors was applied. The plants were then allowed to grow for another 4 days, and then harvested. For the analyses, 10 plants per treatment were randomly chosen, thoroughly mixed, and distributed into four technical replicates. The samples were quickly transported to the laboratory and kept at −80 °C.

### 2.2. Extraction of Intact GSLs

One hundred milligrams of freeze dried, grounded material were extracted with 1 mL of 70% methanol using a water bath at 70 °C for 30 min, with vortex agitation every 5 min. The samples were cooled in an ice bath and centrifuged at 10,000× *g* during 15 min, at room temperature. Supernatants were collected and transferred to a rotary evaporator until the complete removal of methanol. After that, 300 μL of MilliQ water were added. After homogenization, samples were filtered through a 0.22-μm-Ø Millipore filter (Billerica, MA, USA) into vials for HPLC-DAD analysis.

### 2.3. Elaboration of the ITC-Rich Ingredient Prototype 

Free red cabbage aqueous extracts were prepared using freeze dried and ground powder extracted by maceration and agitation using MilliQ water (1:20 w:v), at room temperature and in the dark. Then, the samples were centrifuged at 12,000× *g* for 15 min at room temperature. The supernatants were collected and filtered with an Albet filter. For further storage, aqueous extracts were lyophilized to obtain 50 g batches of freeze dried extracts. Resultant batches were stored at −80 °C.

### 2.4. Microsomal Fraction Extraction 

One hundred milligrams of cauliflower inflorescences (*Brassica oleracea* L. var. botrytis), kindly provided by Sakata Seed Ibérica (S.L.U., Valencia, Spain), were cut into small pieces and vacuum-infiltrated with 0.5 g of PVP and 160 mL of extraction buffer (0.5 M sucrose, 1 mM dithiotreitol (DTT), 50 mM 4-(2-hydroxyethyl)-1-piperazineethanesulfonic acid (HEPES), and 1.30 mM ascorbic acid at pH 7.5. After that, the samples were blended and filtered through a nylon mesh (with 100 μm of pore diameter). The collected filtrate was centrifuged at 10,000× *g* for 30 min, at 4 °C. The supernatants were again centrifuged at 50,000× *g* for 35 min, at 4 °C. The pellet was then resuspended in 500 μL of phosphate buffer (5 mM) with 0.5 M sucrose (pH 6.5).

### 2.5. Plasma Membrane Isolation

Two milliliters of the cauliflower-derived (previously obtained) microsomal fraction were introduced into a two-phase system mixture, with a final composition of PEG-3350/Dextran-T500-6.3% (*w*/*w*), 5 mM KCl, 330 mM sucrose, 2.5 mM NaF, and 5 mM K_3_PO_4_ (pH 7.8). After being centrifuged for 5 min at 4000× *g*, the upper phase was collected and washed with a solution containing 9 mM KCl, 0.2 M EGTA, 0.5 mM NaF, and 10 mM Tris-borate (pH 8.3). Next, the samples were centrifuged at 55,000× *g* for 35 min at 4 °C. Then, 1 g of pellet was resuspended in 1 mL of red cabbage aqueous extract for further sample preparation.

### 2.6. Dynamic Gastrointestinal and Colonic Fermentation Model

Before the colonic fermentation assays, different treatments were prepared: for the non-encapsulated treatment, 1g of red cabbage aqueous lyophilized extract was resuspended and homogenized in 1 mL MilliQ water. For the nanoencapsulated treatment, 1 mg mL^−1^ of red cabbage aqueous extract was nanoencapsulated in 1 mL of cauliflower plasma membrane vesicles. Glycerol was added until obtaining 20 g of total weight, in order to increase the stability of the vesicles during their transport. These samples (1 mL of free red cabbage aqueous lyophilized extract, and 20 mL of nanoencapsulated red cabbage mixture with glycerol) were introduced into the in vitro dynamic gastrointestinal model.

The Dynamic-Colonic Gastrointestinal Digester (D-CGD) was developed by AINIA Technology Center (Valencia, Spain) [41,42,43], which consists of five interconnected double jacket vessels that simulate the physiological conditions of the stomach, small intestine, and the three colonic sections; that is, the ascending colon (AC), transverse colon (TC), and descending colon (DC). The selection of residence times, pH values, temperature (37 °C), and the volume of each reactor were computer-assisted. All the compartments were communicated by peristaltic pumps, working semi-continuously in the stomach and small intestine and continuously for the colonic vessels. Gastric digestion was simulated by continuously adding a 0.03% (*w*/*v*) pepsin solution (2100 units/mg) for 2 h (for a total volume of 60 mL). A typical gastric digestion pH curve (based on in vivo data) was simulated by adding an HCl (1 M) solution. Digestion in the small intestine was mimicked by the continuous addition of a solution containing pancreatin (0.9 g/L), NaHCO3 (12 g/L), and Oxgall dehydrated fresh bile (6 g/L) in distilled water (total volume of 440 mL), maintaining the intestinal content at pH 6.5. In order to maintain anaerobiosis, gaseous N_2_ was flushed for 15 min twice a day. A pool with the feces from four adult volunteers was utilized. The volunteers had dysfunctions or pathologies associated with obesity and/or metabolic syndrome, but had not received any antibiotic treatment during the previous three months, had not consumed enriched foods or supplements with vitamins, probiotics/prebiotics, herbal products, and did not follow weight loss diets or did not have any bowel disease. The inclusion criterion was obesity (34.9 ≥ BMI ≥ 30 kg/m^2^). The exclusion criteria that were considered were: following a weight loss diet, suffering from gastrointestinal diseases (Crohn’s, colitis, IBD,…), clinical diagnosis of cardiovascular diseases, diabetes or cancer, pregnant or lactating women, and current use of drugs (lipid-lowering, antihypertensive, proton-pump inhibitors,…). The diet of the fecal donors was not controlled. The only requisite was that they did not use either antibiotics (in the previous three months) or supplements (probiotics, vitamins, herbs). The objective was to include different phenotypes of obesity-related microbiota patterns in the microbiota sample, a wide age range (22–65 years old), sex (two males/two females), BMI (from 26 to 39), and dietary patterns (from vegetarianism to Western diets, including Mediterranean). With this premise, we intended to obtain a high diversity of microorganisms. The volunteers (of Caucasian ethnicity) were recruited from a population that participated in a previous study from the University of Navarra (Cuevas-Sierra et al., 2020) [44].

A 20% (*w*/*v*) fecal solution was prepared with regenerated thioglycolate, inoculated in the colon compartments (50, 80, and 60 mL for AC, TC, and DC, respectively), and completed with a culture medium up to 1000, 1600, and 1200 mL, respectively. The culture medium was elaborated according to Molly et al. [42,43]. The culture medium provided all the necessary nutritional components to simulate the conditions of the human colon and allow the growth of the intestinal microbiota.

A stabilization period of 11 days was required to allow for the growth of the human fecal microbiota in the colon compartments. This time interval corresponded to the stabilization time required (from 10 to 20 days) to overcome the stabilization of the microbiota (latency period), and to reach a bacterial density that was similar to the colonic environment [45,46]. During this period, 200 mL of culture medium were added to the stomach compartment 3 times a day. Samples from the fermentation liquids (FL) from the AC, TC, and DC compartments, corresponding to time 0, were taken at the end of the microbiota stabilization period. After that, a treatment period of 14 days was started by adding the sample to the in vitro digestion system once per day, and the culture medium twice per day. At the end of the treatment period, the samples were taken from the fermentation liquids from the three compartments. These samples were centrifuged (15,000× *g*, 15 min) and filtered through a 0.22-μm-Ø Millipore filter (Billerica, MA, USA) into vials for UHPLC-ESI-QqQ-MS/MS analysis. The maintenance of the microbial population after the stabilization (time 0) and during the treatment period (time 14) was checked by plate counts of total anaerobic bacteria (on Schaedler agar under anaerobic incubation, 37 °C/48 h) and the other bacterial groups. No relevant differences were observed between both periods (>7 log CFU for the AC, TC, and DC compartments).

In addition, the short and medium fatty acids composition of the fermentation medium was determined according to the relative percentage of chromatographic areas of their corresponding methyl esters. The fat was extracted according to the Folch method (cold extraction) and the esterification of the free fatty acids was carried out using a methanolic potassium hydroxide solution. The methyl esters of the fatty acids were analyzed by gas chromatography coupled to a FID detector. The results were expressed as mg of the compound per kg of fecal medium.

### 2.7. Microbiota Composition and 16S rRNA Analysis

Next Generation Sequencing (NGS) was performed using MiSeq Reagent Kits (Illumina Inc., San Diego, CA, USA). A first PCR was performed on 12.5 ng of genomic DNA obtained from the samples, and 16S-Fw and 16S-Rv primers. After that, a second PCR reaction was performed using 5 μL of DNA and the Nextera^®^ XT DNA Index kit (FC-131-1002, Illumina). Then, the process quality was verified in a Labchip Bioanalyzer (Agilent Technologies Spain S.L., Madrid, Spain). When all the samples were obtained, they were multiplexed by mixing equimolar concentrations from each sample and the internal standard Phix. The mix was diluted until obtaining a concentration of 8 pM. The sequencing was performed in a MiSeq using a MiSeq^®^ Reagent Kit V2 (MS-102-2003).

### 2.8. Bioinformatic Analysis

The 16S rRNA sequences obtained were curated following the quality criteria from the OTUs processing protocol, using the LotuS pipeline [47] This protocol includes the clustering of de novo sequences by UPARSE and the deleting of chimeric and contaminant sequences for OTUs identification. In addition, this program generates the corresponding abundance matrix. An OTU is defined as organisms that are clustered according to the similarity of their DNA sequence. The taxonomy was assigned by using BLAST and HITdb, reaching a species sensitivity level. The abundance matrix was curated and normalized in R and Bioconductor. A global normalization was performed using the library size as a correction factor. Data was transformed to Log2.

### 2.9. Glucosinolates and Isothiocyanates Quantitative Determination

Glucosinolates extracted from fresh red cabbage were identified by HPLC-DAD-ESI-MS^n^, according to their [M-H] and MS^2^ fragmentation patterns. The conditions for analysis were the same as described in Baenas et al. [48]. For the quantitative analysis of intact glucosinolates, 20 μL of extract were introduced into an Agilent 1100 HPLC-DAD system (Santa Clara, CA, USA). The glucosinolates were identified according to their UV spectra and elution order. Sinigrin and glucobrassicin were used as external standards (Phytochem, Neu-Ulm, Germany). Isothiocyanates were measured by a high throughput UHPLC-QqQ-MS/MS method, as described in Baenas et al. [49]. The standards employed for quantification were sulforaphane (SFN), SFN-glutathione (SFN-GSH), SFN-cysteine (SFN-CYS), SFN-N, acetylcysteine (SFN-NAC), iberin, and indole-3-carbinol (I3C) from Santa Cruz Biotechnology (Dallas, TX, USA), via Quimigen S.L. (Madrid, Spain).

### 2.10. Data Analysis 

For the field elicitation experiment, a one-way ANOVA was performed, using Tukey’s HSD as a *post hoc* test. For the dynamic digester experiments, a two-way ANOVA was applied, also followed by Tukey’s HSD as a *post hoc* test. All of these analyses were carried out in RStudio (version 3.6.3).

## 3. Results

### 3.1. Field Elicitation of Red Cabbage

Six different glucosinolates were identified in the red cabbage samples: glucoiberin (GIB), glucoraphanin (GRA), sinigrin (SIN), gluconapin (GNA), 4-hydroxy-glucobrassicin (HGB), and glucobrassicin (GB). The HPLC-DAD analyses allowed the quantification of the three major compounds together with the study of the effect of the different elicitors (Table 1) on the red cabbage GSLs. The concentration of SIN significantly decreased after the application of 200 μM SA (*p* < 0.05). No differences were found between 100 μM MeJA and the combination treatment, when compared with the control (*p* > 0.05). The results for indolic HGB were similar, in that the 200 μM SA treatment decreased its content (*p* < 0.05), but 100 μM MeJA significantly increased it (2-fold) (*p* < 0.05). The combination treatment also reduced the HGB content (*p* < 0.05). Similarly, the GB was not affected by the 200 μM SA (*p* < 0.05) treatment, but increased dramatically with the other treatments: 3-fold with the 100 μM MeJA treatment, and 2-fold in the combination treatment. The total GSLs content followed the response observed in the individual compounds, and the 200 μM SA treatment decreased its value (*p* < 0.05), although the 100 μM MeJA (2-fold) and the combination treatment (1.5-fold), were very positive, when compared to the untreated controls.

### 3.2. Characterization of Extracts

The results presented in Table 2 showed that no statistically significant differences were obtained between treatments for SFN, I3C, or iberin (*p* > 0.05). The absence of GSLs was also corroborated by the HPLC-DAD analysis of the samples.

### 3.3. Dynamic Gastrointestinal and Colonic Fermentation Model

**Figure 1:**
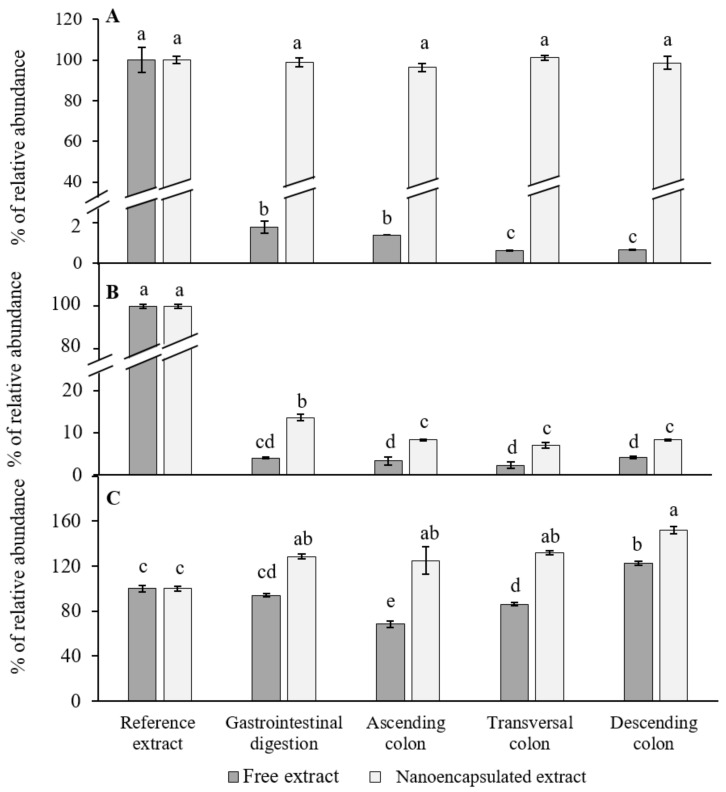
Percentage of the relative abundance of (**A**) sulforaphane (SFN), (**B**) indole-3-carbinol, and (**C**) iberin, when feeding the dynamic colonic-gastrointestinal digester (D-CGID) with the red cabbage aqueous extract, both free and nanoencapsulated (*n* = 3 ± SE). The reference extract was taken as 100% and a two-way ANOVA analysis with an HSD Tukey’s test as a *post hoc* test was performed. Different letters indicate statistically significant differences (*p* < 0.05).

The presence of ITCs was clear in the two types of samples, free and encapsulated (Figure 1A), and more importantly, regarding the digestion treatment with the aqueous (free) extract, 1.77% of the starting concentration was still available after the gastrointestinal (GI) digestion process. No statistically significant differences were found when comparing the GI digestion with the ascending colon fermentation (*p* > 0.05). However, a decrease in SFN (from 14% to 6% from the initial dosage) was observed when comparing the ascending colon reactor with the transversal and descending ones (*p* < 0.05). With respect to the nanoencapsulated extract, the percentage of bioaccessible SFN did not decrease after GI digestion (retention of 99.4%), and no variations were observed in the three reactors of colonic fermentation (*p* < 0.05). When comparing both treatments, the nanoencapsulated red cabbage aqueous extracts showed the highest percentages of abundance of SFN (*p* < 0.05).

Regarding indole-3-carbinol (I3C, Figure 1B), when analyzing the free red cabbage aqueous extract digestions, 4% remained present after the digestion process (*p* < 0.05). No statistically significant differences were found when compared with the contents after the colonic fermentation in the three reactors (*p* > 0.05). On the other hand, from the nanoencapsulated red cabbage aqueous extract, a higher retention of I3C (by 12%) was observed after the GI digestion when compared with the crude extract (*p* < 0.05), and a statistically significant decrease (1.5-fold) was found after passing through the ascending colon reactor (*p* < 0.05). Nevertheless, no differences were found between the three colonic reactors (ascending, transversal, and descending colon) (*p* > 0.05).

As for the presence of iberin (Figure 1C), no statistically significant decreases were observed after GI digestion (*p* > 0.05). However, when compared with the ascending colon reactor, a 1.5-fold decrease was observed (*p* < 0.05), with an increase observed in the transversal and descending colon (1.5 and 2-fold, respectively) (*p* <0.05). In addition, the final retention of iberin increased in the descending colon reactor as compared to the initial dosage (*p* <0.05). Lastly, with respect to the nanoencapsulated form, a significant increase was observed between the GI digestion and the starting extract (*p* < 0.05). However, no differences were observed between the GI digestion and the three reactors of colonic fermentation (*p* > 0.05).

### 3.4. Effects of the Red Cabbage Extracts on the Microbiome

**Figure 2:**
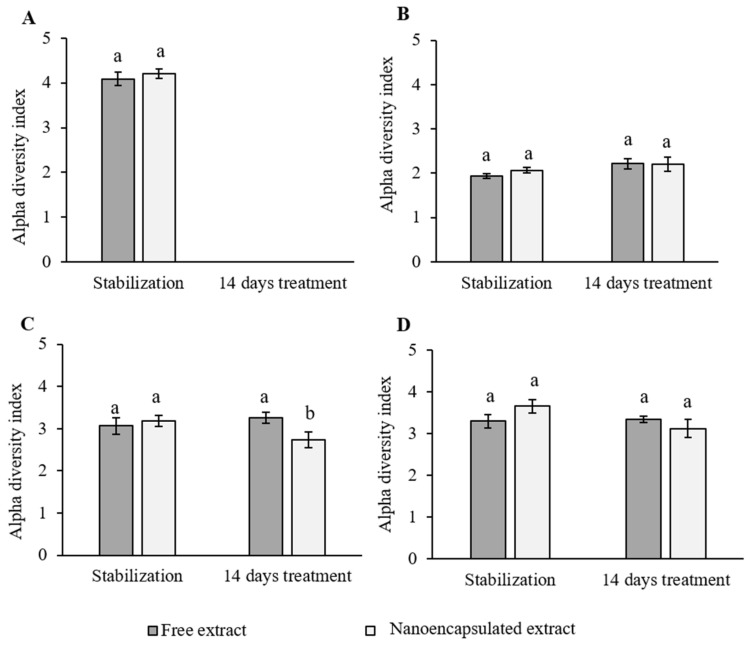
Representation of the alpha diversity index obtained from the inoculation (**A**) and each reactor: (**B**) ascending colon, (**C**) transversal colon, and (**D**) descending colon, before and after the treatment for 14 days with the red cabbage aqueous extract in its free and nanoencapsulated forms (*n* = 3 ± SE). Data were analyzed with a two-way ANOVA and the HSD Tukey’s test as a *post hoc* test. Different letters mean statistically significant differences (*p* < 0.05).

As shown in Figure 2A, no differences were found between the alpha index of the two treatments’ inoculations (*p* > 0.05). For the ascending colon (Figure 2B) no differences were found between the stabilization and the 14-day treatment, or between treatments. In the transversal colon, a statistically significant decrease (*p* < 0.05) in the alpha index was observed after the 14-day treatment with the nanoencapsulated treatment (Figure 2C). Similar results as observed in the ascending colon were observed in the descending colon reactor (Figure 2D, *p* > 0.05).

**Figure 3:**
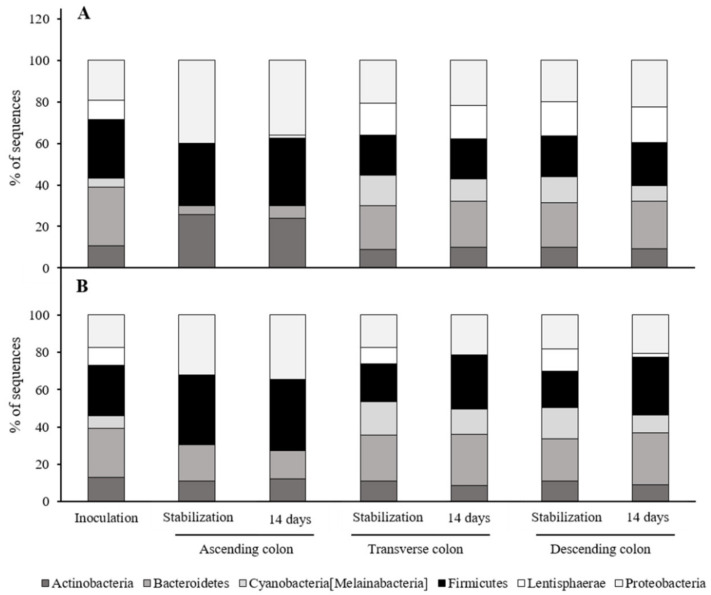
Relative presence (% of sequences identified) of the six most abundant phyla in the human colon in the dynamic in vitro gastrointestinal digestion and colonic fermentation model when fed with (**A**) free red cabbage aqueous extract, and (**B**) nanoencapsulated red cabbage aqueous extract. Data were obtained from the stabilization period (no treatment) and after 14 days of treatment. Represented data are means ± SD. Different letters mean statistically significant differences in the HSD Tukey’s test (*p* < 0.05).

The effect of the treatments on the percentage of relative sequences, from the six most-relevant phyla present in the human microbiome obtained from obese subjects, was analysed. For both fermentation processes, the percentage of sequences was similar at the inoculation point for the six phyla: 10–12% Actinobacteria, 28–26% Bacteroidetes, 4–7% Cyanobacteria, 28–27% Firmicutes, 9.5–9.6% Lentisphereae, and 19–17% Proteobacteria (Figure 3A,B). As for the assay performed with the free red cabbage aqueous extract, the ascending colon stabilization lacked the representation of the Cyanobacteria and Lentisphareae phyla (Figure 3A). However, 1.7% of the sequences corresponding to Lentisphaerae was detected when the treatment was applied after 14 days. In regard to the assay performed with the nanoencapsulated red cabbage aqueous extract (Figure 3B), sequences from four phyla were identified in the ascending colon: Proteobacteria, Firmicutes/Bacteroidetes/Actinobacteria. Nevertheless, no sequences related with the Lentisphaerae phyla or Cyanobacteria were found either at the end of the stabilization period or after the treatment. The samples from the analysed transverse colon (Figure 3B) showed sequences belonging to the six most relevant phyla after the stabilization treatment, but no sequences corresponding to the Lenstisphaerae phylum were identified after 14 days of treatment with the nanoencapsulated red cabbage aqueous extract. According to the descending colon, sequences corresponding with the six main phyla were identified in both the stabilization process and after the treatment. However, the percentage of sequences corresponding to Firmicutes varied from 19% in the stabilization process to 31% after the treatment with the nanoencapsulated red cabbage aqueous extract (Figure 3B). The relative percentage of sequences identified with Lentisphaerae and Cyanobacteria also differed after the treatment, from 11.7% to 1.5% for the first phylum, and from 16.7% to 9.6% for the second.

The *Bacteroidetes/Firmicutes* ratio (B/F ratio) was also obtained for both fermentation assays (Table 3). No statistically significant differences were observed for the inoculation in both treatments (*p* > 0.05). For the ascending colon, only a decrease between the stabilization and the 14-day treatment was observed when using the nanoencapsulated treatment (*p* < 0.05). Similar results in the B/F ratio were observed for the transversal and the descending colons, showing a decrease after the nanoencapsulated treatment (*p* < 0.05).

### 3.5. Butyric Acid Production by the Microbiota

**Figure 4:**
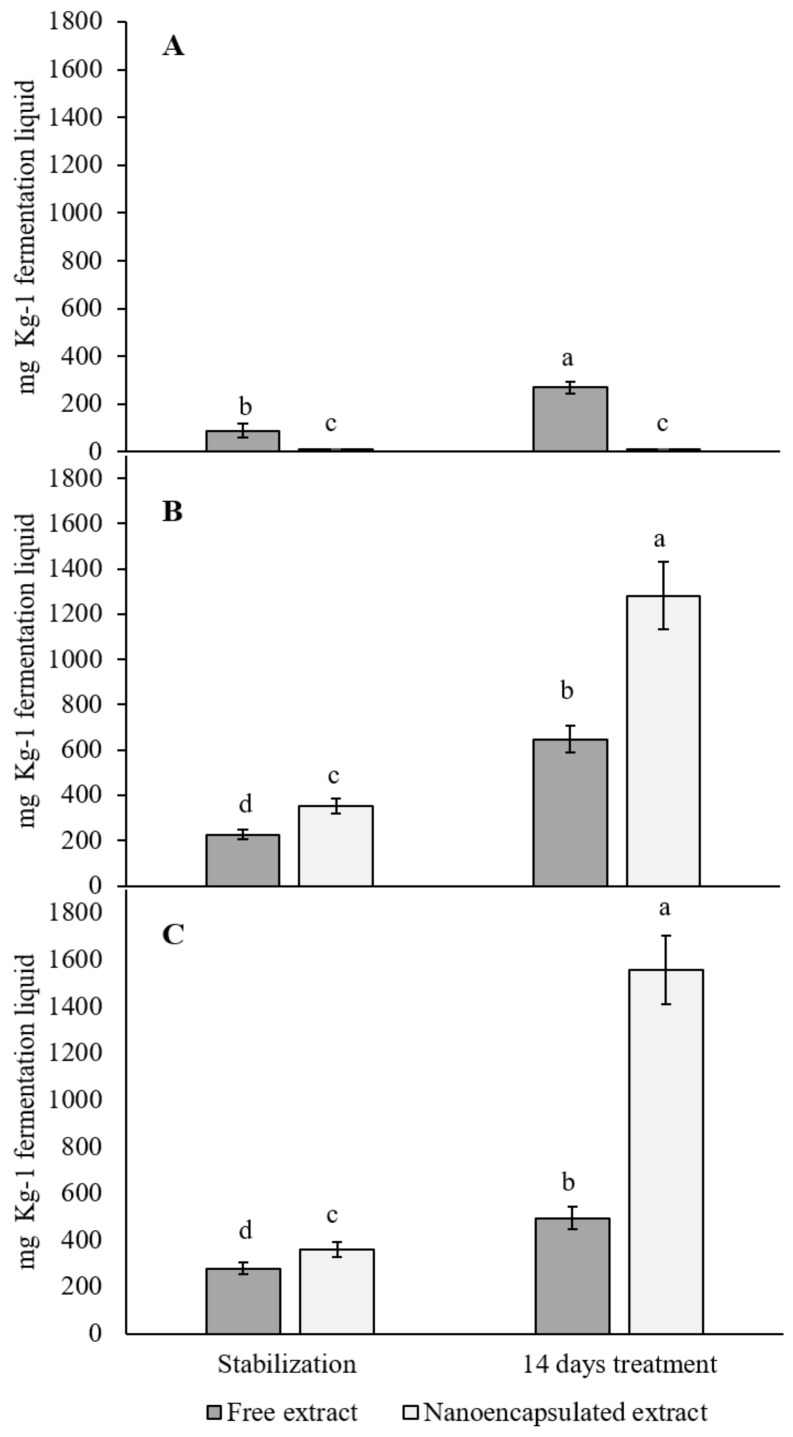
Butyric acid production (mg·Kg^−1^ fermentation fluid) analysed in the stabilization period and after 14 days of treatment with the red cabbage aqueous extract (free and nanoencapsulated) in the (**A**) ascending colon, (**B**) transversal colon, and (**C**) descending colon. Represented data are means ± SD. Different letters mean statistically significant differences in the HSD Tukey’s test (*p* < 0.05).

The butyric acid production by gut microbiota was analysed in the three digester reactors (mg·Kg^−1^ fermentation liquid). For the ascending colon reactor, an increase in its production was observed after the 14-day treatment with the free red cabbage aqueous extract (Figure 4A, *p* < 0.05). No statistically significant differences were observed with the nanoencapsulated treatment (*p* > 0.05). As for the transversal colon reactor (Figure 4B), a 3-fold increase after the 14-day treatment was observed for the free red cabbage aqueous extract when compared to the stabilization period (*p* < 0.05). Additionally, a 3.5-fold increase was observed when using the nanonencapsulated extract (*p* < 0.05). Regarding the descending colon (Figure 4C), similar results as observed in the transversal colon were obtained. A 2-fold increase was produced after 14 days of treatment with the free red cabbage aqueous extract (*p* < 0.05). In addition, a statistically significant increase (4-fold) was observed in the production of butyric acid after the treatment with the nanoencapsulated extract (*p* < 0.05). Lastly, propionic and acetic acids were studied, but no changes were observed (*p* > 0.05, data not shown).

## 4. Discussion

At present, there is a growing interest in functional foods and beverages obtained from vegetables, as they concentrate bioactive compounds and are considered less time-wasting by health-conscious consumers [18]. However, good quality starting plant material is required for creating a formula enriched in bioactive compounds. For this purpose, the use of elicitors on red cabbage to improve the contents of GSLs was positive under field conditions, as studied here. Our results showed that a foliar application with 100 μM MeJA greatly increased the concentrations of indolic GSLs (HGB and GB; Table 1). Previous works [50] found the elicitation with 200 μM jasmonic acid as positive for red cabbage, but with only an increased content of SIN. However, in our work, SIN remained unaffected. In Hassini et al. [51], MeJA elicitation on red cabbage sprouts did not report increases in SIN, but dramatically increased GB (7-fold increase). Jasmonic acid and its derivatives (such as MeJA) are known to mainly increase the accumulation of indole GSLs [15,16,17,52], due to their key role as regulator in the jasmonic acid-signaling pathway *MYB34* [53]. Our experimental design under field conditions, the application protocol, as well as the harvesting time, were all factors involved in the response of the plant to the treatments. Similar results were found for GB in kale and cabbage after MeJA elicitation [53], but also with an increased content of GSLs when using SA; as opposed to our findings, which agreed with the work by Thiruvengadam et al. [54], where decreases in HGB were also observed after 100 μM SA spray applications to turnips. Therefore, the results of the elicitation using these compounds as inducers vary depending on the species under study. The performance of the elicitor is not only based on the elicitor–species relationship, but such interaction also plays a differential role under different growing conditions.

Once a GSLs-enriched plant material was obtained, we opted for an aqueous extract enriched in ITCs from red cabbage, since hydrodistillation is more environmentally-friendly, more compatible with a food grade product than the use of organic solvents, and more representative of a dietary approach for the use of the bioactive compounds in a nutritional intervention.

Different types of stabilization methods for ITCs have been studied up to the present, for example, using cyclodextrins in an inclusion complex, or chitosan as coating material [55,56]. However, the coating or wall material is usually very expensive, restricting this type of carrier to high value-added industries, such as the pharmaceutical industry. In this way, ITCs encapsulation for stabilization in other industries still remains a field to be further investigated. In our work, a nanoencapsulation method based on plasma membrane vesicles obtained from cauliflower inflorescences [29] was studied. The use of plant-derived plasma membrane vesicles has been widely studied by our group and provided very positive results in different fields. For example, when used as nanobiofertilizers along with iron and boron for almond trees, a great increase in leaf concentration was observed [57]. Moreover, their potential in the dermatological industry has been assessed in skin keratinocytes, demonstrating their delivery and high penetrability [30]. These previous studies have led us to evaluate cauliflower-derived plasma membrane vesicles as ITCs stabilizers in a food prototype.

On the other hand, before arriving to their target cells or tissues, ITCs should be extracted from a complex matrix during digestion, and then absorbed by the small intestine [58]. For that purpose, a dynamic in vitro gastrointestinal digestion model was employed to estimate bioaccessibility. This in vitro digestion model offers a high reproducibility, ease of control and handling, and can provide a great approach to further in vivo experiments [59]. As for bioaccessibility, it is defined as the quantity of a compound present in food that is released from the digestive bolus into the GI tract, becoming available for absorption [60]. Regarding our results, the SFN analysis from the digestion simulation performed with the free red cabbage aqueous extract, revealed a dramatic decrease in its content after GI digestion (*p* < 0.05, Figure 1A). Xiangang et al. [61] reported on the high stability of SFN obtained from fresh broccoli seeds and sprouts after performing an in vitro GI digestion. This is in contrast with our results, suggesting that the food matrix plays a key role in the further extraction of bioactives during the digestion process. In this way, when the plasma membrane vesicles were added to the red cabbage aqueous extract, a high percentage of SFN was conserved through the gastrointestinal digestion (Figure 1A). The work by Martínez-Ballesta et al. [62] reported a putative interaction by molecular docking between some proteins (aquaporins) present in the nanocapsules (nanovesicles enriched in aquaporins) obtained from broccoli plants and the glucosinolate glucoraphanin, which increased molecule stability. Therefore, there could also be an interaction between the plant aquaporins found in our vesicles [29] and the ITCs present in the red cabbage aqueous extract, which could have increased stability. In this way, plasma membrane vesicles may act as stabilizing carriers and feeding agents for enzymes and bile salts rather than an encapsulating agent *per se*. However, this aspect should be further studied.

As for I3C (Figure 1B), its stability also increased by the presence of cauliflower-plasma membrane vesicles, with a higher percentage remaining after the gastric digestion (*p* < 0.05). The stability of I3C is known to be a major problem when studying its bioavailability, as these molecules undergo oligomerization and form a mixture of diverse acid condensation products under acidic conditions [63]. Although encapsulation with zein reported an increase in I3C stability against thermal treatments, little is known about its performance under low pH conditions [64]. Regarding iberin (Figure 1C), it has been reported that it is usually more stable under acidic pH conditions. This could explain the high preservation of iberin even in the free red cabbage aqueous extract after the gastrointestinal digestion. Furthermore, for the nanoencapsulated extract, an increase in its relative abundance was observed after the gastrointestinal digestion. An explanation for this phenomena could be that after 14 days of treatment, an accumulation of iberin took place, as it has a low hydrophobicity, and the interaction with the proteolipidic vesicles may have stabilized it through time [65]. Furthermore, the high increase observed in the nanoencapsulated treatment after the colonic fermentation may also be due to the myrosinase activity of some enzymes from the colonic bacteria [66]. In addition, although many studies exist on the bioconversion between glucoraphanin and SFN, and its bioaccessibility [61,67,68], little information is available about the performance of other ITCs under GI digestion and colonic fermentation.

In the last decade, the high importance of the gut microbiome has been brought to light. Many studies which focused on fecal transplants revealed that the disturbance of gut microbiota is involved in the pathogenesis of NCDs [69,70,71]. Therefore, adjuvants such as specific diets, probiotics and prebiotics, and fecal transplants emerged as new approaches for modulating the gut microbial ecosystem [72]. Usually, the term dysbiosis is employed to define the disturbance of the relative abundance of the microbial groups, often classified by the sequencing of the 16S rRNA [73]. Nevertheless, it is not clear what a “dysbiotic” or “healthy” profile is, as a specific profile could be dysbiotic for an individual, but the same profile could describe a healthy subject [74]. Even though there is extensive discussion on this topic, we wanted to assess the effect of our red cabbage aqueous extracts (free and nanoencapsulated) during 14 days of treatment on the gut microbiome obtained from obese volunteers.

One of the main parameters that provides information about a microbiome is the alpha diversity index, as it describes the species richness based on the OTU count [75]. Our study did not show large variations in the alpha index between the stabilization period and after the 14-day treatments (Figure 2). Similar results were observed by Kazmarek et al. [8], where no significant changes were observed in their alpha index after an 18-day treatment with a broccoli-based diet consumed by human subjects. In addition, our results showed a higher diversity in the transversal and descending colon, whereas the lowest alpha values were obtained in the ascending colon (Figure 2). In brief, it seems that our extracts neither affected the species richness nor caused the extinction of OTUs, but rather decreased them. SFN has been reported to have anti-microbiological effects against gut pathogens in vitro [58]. Since our red cabbage-derived extracts were rich in SFN, the 14-day treatments might have affected not only the gut pathogens present in the inoculus, but also the beneficial bacteria populations, thus decreasing the OTUs count.

The relative presence of the six most abundant phyla analysed in our experiment was also determined (Figure 3). Among these phyla, we found the four major bacterial groups reported by the bibliography to colonize the adult human gut: Bacteroidetes (Gram-negative anaerobes), Firmicutes (Gram positive), Actinobacteria (Gram positive), and Proteobacteria (Gram negative) [76]. Although they have been described as less abundant [77], Lentisphaerae and Cyanobacteria phyla were also reported as some of the most representative in our study. Our results did not report differences between the stabilization period and the treatment after 14 days when applying the two treatments (Figure 3). Since it has been reported that dietary interventions could cause a shift in the gut microbiome within 24 h, perhaps the time set for our analysis was too long [78]. Nevertheless, some changes were observed after the treatments: in the ascending colon, the percentage of sequences belonging to the phylum Lentisphaerae (Figure 3A) increased from 0% to 1.67% after the treatment with the free red cabbage aqueous extract. In addition, a decrease in the percentage of sequences identified as Cyanobacteria was observed in the transversal (14% down to 10%) and descending colons (13% down to 8%). A similar result was observed for Cyanobacteria when the nanoencapsulated treatment was applied (Figure 3B); a decrease from 17% to 13% in the transversal colon, and a decrease from 17% to 9% in the descending colon. Recently, these phyla have been positively associated with the low-density lipoprotein cholesterol (LDL-C), which decreases the effect of rosuvastatin in patients with hyperlipidemia [77]. Although the conditions were not similar, the decrease in the Cyanobacteria group observed in our work may have a health-related influence on the blood cholesterol levels of obese patients. Proteobacteria are currently the largest phylum in the bacterial domain and are Gram negative, which means that they contain lipopolysaccharides in the outer membrane. Thus, a putative deleterious role has been associated with a Proteobacteria bloom and intestinal inflammation due to the increase in lipopolysaccharide levels [79]. Furthermore, it has been reported that the monocolonization of a germen-free mouse gut with *Enterobacter cloaceae* B29 induced obesity in its host. Therefore, Proteobacteria have been defined as putative gut dysbiosis markers [80]. Nevertheless, in our study, only subtle changes were found in the Proteobacteria phylum when the treatments were applied. For the free red cabbage aqueous extract (Figure 3A), a decrease at the ascending colon was observed after the treatment (40% vs. 35%). Regarding the nanoencapsulated treatment (Figure 3B), a slight increase was found in the transversal (17% vs. 20%) and descending (18% vs. 21%) colons when the nanoencapsulated treatment was applied. Similar results have been reported in rats’ gut microbiome after broccoli ingestion, in which a decrease in the Proteobacteria population was observed [81].

According to experiments performed by Ridaura et al. [82], microbial inoculation from an obese model into a lean twin resulted in a progressively greater increase in fat mass and body weight. Hence, many efforts have been made to pinpoint what composes a “healthy” and “obese” microbiome. Derived from these works, an obese phenotype has been linked to a lower *Bacteroidetes/Firmicutes* ratio, whereas this ratio increases in lean phenotypes [83]. In our study, the *Bacteroidetes/Firmicutes* ratio remained close to the one found in the inoculation and in the transversal and descending colon reactors, even after the treatments with the red cabbage aqueous extracts (free or nanoencapsulated). Only in the ascending colon were the ratios obtained below 0.5. Since the studies performed in humans usually did not focus on the three parts of the colon separately, perhaps this colon compartment is a better niche for *Firmicutes*. An increase in the *Bacteroidetes* phyla has been reported in obese mice models after being fed with rice bran [84]. Furthermore, probiotics such as *Lactobacillus salivarius* Ls-33 have been observed to increase the *Bacteroidetes/Firmicutes* ratio in obese adolescents [85]. Nevertheless, this association is not exempt from controversy, as other studies have criticized this ratio for its lack of specificity [86]. For example, recent research performed by Cortés-Martín et al. [87] revealed that no distinctive gut microbiota signature was found related with metabolic syndrome after analysing samples from 69 obese volunteers and 50 patients with metabolic syndrome. On the other hand, authors such as Aoun et al. [88] manifested that the gut microbiome has an influence in the host nutrient metabolism and energy expenditure, suggesting that further clinical studies are needed to understand how species affect weight gain. In brief, since the influence of the gut microbiome on obesity not only underlies the colon bacteria, but also interconnects genetics, the environment, the immune system, and the brain, it is highly complicated to decipher the exact consequences on metabolic alterations.

Lastly, for butyric acid production, short chain fatty acids (SCFAs) are products that mainly come from the microbes performing anaerobic metabolism of non-digestible carbohydrates. Butyric acid has been reported to regulate the immune function in the intestine [89]. However, recent studies have shown that butyric acid is able to interact with the orexigenic neurons present in the hypothalamus, whose role is to mediate food intake and provide a protective effect against the effects of a high-fat diet [90]. Furthermore, a lower presence of butyrate-producing bacteria has been linked with an increased risk of metabolic disease [91]. A study was conducted with obese volunteers suffering from metabolic syndrome, who were administered with sodium butyrate (4 g/day), showing a positive anti-inflammatory and immunomodulatory effect [92]. As observed in our results, an increase in butyrate production by the gut microbiome was observed for both treatments (*p* < 0.05), although this was significantly higher with the nanoencapsulated extract. In this way, our treatments might exert an effect on the butyrate-producing bacteria taxa, such as *Roseburia intestinalis*, *Faecalibacterium prausnitzii*, or *Eubacterium rectale* [89]^.^ As the microbiome was obtained from obese volunteers, the improved production of this SCFAs may have a direct effect on the food intake by modulating the afferent neurons in the vagal nervous system [93]. Further in vivo research should be conducted to prove the direct effect of the increase in the SCFAs produced by the intake of our extracts on obese subjects and the effect of the treatments on the butyrate-producing bacteria.

## 5. Conclusions

The present comprehensive study, from plant to food ingredient and health, evaluated the potential for the red cabbage encapsulated prototype enriched in GSL to improve its bioaccessibility for gastrointestinal absorption. This was conducted after evaluating its composition, and with improved stability of its ITCs by encapsulation with plant membrane vesicles. The results showed that the encapsulation helped with reaching the GI microbiota, with the interest placed on modulating the interaction between phyla to improve the metabolic and energy state of human adults suffering from overweightness and obesity. Furthermore, the fact that the encapsulated red cabbage extract provided a higher production of butyric acid, pointed to future developments for the design of a functional ingredient or food product for the management of overweightness and obesity in the adult population. This is of great interest to the public health systems worldwide because of its pandemic proportions. The search for natural alternatives to medications, to be incorporated into diets and nutritional interventions, and to help in the management of overweightness and obesity, has garnered interest worldwide and will have a global impact. However, there is a current need for further research on the bioavailability, metabolism, and bioactivity of natural plant-based products.

## Figures and Tables

**Table 1:** Effect of elicitors on glucosinolates of red cabbage inflorescences. The numbers show the average values per treatment (*n* = 4) ± standard error. Different letters in a row indicate statistically significant differences in the HSD Tukey’s test (*p* < 0.05).

Glucosinolate (mg g D.W.^−1^)	Control	200 μM SA	100 μM MeJA	SA + MeJA
GIB	*	*	*	*
GRA	*	*	*	*
SIN	4.12 ± 0.13 a	3.73 ± 0.08 b	4.95 ± 0.04 a	4.12 ± 0.06 ab
GNA	*	*	*	*
HGB	0.77 ± 0.02 b	0.41 ± 0.03 c	1.79 ± 0.02 a	0.54 ± 0.02 c
GB	2.93 ± 0.05 c	2.24 ± 0.08 c	8.1 ± 0.06 a	5.01 ± 0.1 b
Total GSLs	8.13 ± 0.2 b	6.39 ± 0.03 c	14.82 ± 0.01 a	9.71 ± 0.08 b

SA: salicylic acid, MeJA: methyl jasmonate, GSL: glucosinolate, GIB: glucoiberin, GRA: glucoraphanin, SIN: sinigrin, GNA: gluconapin, HGB: 4-hydroxy-glucobrassicin, GB: glucobrassicin. * The presence of the GSLs was under the limit of quantification for HPLC-DAD-ESI-MS^n^ (<0.02 mg g D.W.^−1^).

**Table 2:** Extract composition of the red cabbage aqueous extract for the free and the nanoencapsulated treatment. The numbers show the average values (*n* = 3) ± standard error. Different letters in a row indicate statistically significant differences in the HSD Tukey’s test (*p* < 0.05).

	Red Cabbage Aqueous Extract	
ITCs Composition (μg/mL)	Free Extract	Nanoencapsulated
Sulforaphane (SFN)	6.72 ± 0.68 a	5.64 ± 0.28 a
Indole-3-carbinol (I3C)	1.81 ± 0.15 a	1.05 ± 0.1 a
Iberin	1.82 ± 0.04 a	1.97 ± 0.06 a

ITCs: isothiocyanates.

**Table 3:** *Bacteroidetes/Firmicutes* ratio obtained in the inoculation time and in the ascending, transversal, and descending colon reactors after the feeding with red cabbage aqueous extract (free or nanoencapsulated) for 14 days. The results show *n* = 3 ± SE. Different letters indicate statistically significant differences in the HSD Tukey’s test, performed after a two-way ANOVA (*p* < 0.05).

*Bacteroidetes/Firmicutes* Ratio	Free Red Cabbage Aqueous Extract		Red Cabbage Nanoencapsulated Aqueous Extract	
	Stabilization	14 Days Treatment	Stabilization	14 Days Treatment
Inoculation	1.00 ± 0.02 a	-	0.98 ± 0.07 a	-
Ascending colon	0.15 ± 0.01 c	0.19 ± 0.08 c	0.5 ± 0.03 a	0.39 ± 0.02 b
Transversal colon	1.08 ± 0.1 ab	1.14 ± 0.08 a	1.21 ± 0.16 a	0.95 ± 0.05 b
Descending colon	1.08 ± 0.03 ab	1.09 ± 0.04 ab	1.16 ± 0.08 a	0.90 ± 0.01 b

## Data Availability

Data supporting reported results and generated during the study are available upon requesting them from the corresponding author.
